# Characterization of the Complete Mitochondrial Genome of *Cricula andrei* (Lepidoptera: Saturniidae) and Comparison with Other Lepidoptera Species

**DOI:** 10.3390/cimb48070741

**Published:** 2026-07-21

**Authors:** Xiangrong Meng, Wentao Yang, Yuan Liu, Yongqi Zhang, Die Luo, Siyu Wei, Yifan Guo, Cen Qian

**Affiliations:** 1 College of Life Sciences, Anhui Agricultural University, Hefei 230036, China; 22113725@stu.ahau.edu.cn (X.M.); 24720608@stu.ahau.edu.cn (W.Y.); liuyuan202405@163.com (Y.L.); 19114184@stu.ahau.edu.cn (Y.Z.); 25720409@stu.ahau.edu.cn (D.L.); 050507@stu.ahau.edu.cn (S.W.); 050401@stu.ahau.edu.cn (Y.G.); 2 Anhui Province Key Laboratory of Resource Insect Biology and Innovative Utilization, Anhui Agricultural University, Hefei 230036, China

**Keywords:** mitochondrial genome, lepidoptera, *Cricula andrei*, phylogenetic analysis

## Abstract

As the second largest order of Insecta, Lepidoptera is an important component of the ecosystems. However, there are few data on the mitochondrial genomes of Saturniidae. Here, the complete mitochondrial genome of *Cricula andrei* was sequenced and characterized. It was 15,324 bp in length, containing 13 protein-coding genes (PCGs), 22 tRNAs, 2 rRNAs and a control region (AT-rich region). The control region had an AT content of 90.40%, and the conserved ATAGA sequence guided a 19 bp poly-T. Amino acid composition analysis showed that Ile, Leu, Phe and Asn were the most frequent amino acids, and codon usage analysis revealed a preference for A/U-ending codons. Phylogenetic trees constructed by Bayesian inference and Maximum likelihood methods indicated that *C. andrei* is grouped with *C. trifenestrata* and supported the current taxonomic placement of *Cricula* within the family. The enrichment of the mitochondrial genome database of Lepidoptera will help to better understand the genetic and evolutionary relationships of lepidopteran populations, as well as related taxonomic issues.

## 1. Introduction

As a semi-autonomous organelle, the mitochondrial DNA (mtDNA) is an ideal molecular marker for studying species classification, evolutionary relationships and population genetics because of its simple structure, maternal inheritance, high mutation rate and relatively conservative characteristics compared with the nuclear genome [1,2,3,4,5,6,7].

Insect mitochondrial genome data have exploded in recent years with the widespread use of next-generation sequencing technologies [8]. The analysis of the mitochondrial genome structure of insects can provide basic information for their taxonomy, phylogeny, population genetics, adaptive evolution and diversity [9,10,11,12]. Insect mitochondrial genomes are generally 14–20 kb in size, and are composed of double-stranded closed circular DNA, namely majority strand (J strand) and minority strand (N strand) [1]. Most genes are distributed on the J strand, while a few genes are distributed on the N strand. Insect mitochondrial genomes are conserved and consist of 13 protein coding genes (PCGs), 22 transfer RNAs (tRNAs), 2 ribosomal RNAs (rRNAs) and a control region rich in AT bases (AT-rich region), and only a few insects have the phenomenon of individual gene deletion [2,3,8].

Lepidoptera is the second largest order of Insecta, which contains more than 200,000 species. As a key component of the ecosystems, there are not only pests closely related to human beings, such as *Cnaphalocrocis medinalis*, *Helicoverpa armigera*, *Spodoptera exigua* and *Plutella xylostella*, etc., but also resource insects with important economic value, such as silk insects and pollinators. At present, the Genbank database of NCBI contains thousands of mitochondrial genome sequences of Leidoptera insects, more than half of which are concentrated in Noctuoidea and Papilionoidea, but only approximately 30–40 records forSaturniidae and Bombycidae.

Molecular phylogenetic analyses have become essential tools for investigating evolutionary relationships and taxonomic issues among Lepidoptera. Bayesian inference (BI) and Maximum likelihood (ML) are two widely used model-based approaches for reconstructing phylogenetic relationships. ML estimates the phylogenetic tree by identifying the topology that best explains the observed sequence data under a specific evolutionary model, whereas BI calculates the posterior probability of alternative phylogenetic hypotheses by combining prior information with sequence likelihoods [13,14]. Both methods have been extensively used in mitochondrial genome-based studies to resolve phylogenetic relationships and clarify evolutionary patterns within Lepidoptera [12,15,16,17,18,19,20].

*Cricula andrei* (Jordan, 1909) belongs to the genus *Cricula* in the family Saturniidae, which is mainly distributed in Sichuan, Guangdong, Hainan, Yunnan and Tibet of China, as well as Indonesia. Its larvae feed on the leaves of peach, apple, pear and other plants. Although it has occasionally been reported as a foliage-feeding pest, its economic impact is generally considered limited. However, accurate species identification and knowledge of its evolutionary relationships remain important for biodiversity assessment and for monitoring populations in regions where it occurs. Mitochondrial markers are widely used for species discrimination, population genetic analyses, monitoring of species dispersal, and reconstruction of evolutionary relationships due to their conserved gene content and relatively rapid evolutionary rates. Moreover, the biological characteristics and molecular information of *C. andrei* remain poorly understood.

Here, the complete mitochondrial genome of *C. andrei* was analyzed, and its structural characteristics, base composition, PCGs, rRNA and tRNA genes, the control region and codon usage bias were discussed. At the same time, the mtDNA sequences were compared with those of other lepidopterans, and the phylogenetic trees were constructed by Bayesian inference (BI) method. This study will enrich the mitochondrial genome database of lepidopterans and provide valuable molecular markers for species identification, phylogenetic reconstruction, comparative genomics and population genetic studies.

## 2. Materials and Methods

### 2.1. Experimental Insects, Mitogenome Amplification and DNA Sequencing

*Cricula andrei* were collected from the suburbs of Xi’an City, Shaanxi Province, China, on 30 May 2021. A total of three adults were collected and the voucher specimens were deposited in the School of Life Sciences, Anhui Agricultural University. The collected specimens were identified by taxonomists from the Department of Entomology of Anhui Agricultural University in China based on morphological characteristics as *C. andrei*. The identification was further confirmed through detailed examination of diagnostic morphological traits and comparison with data in the reference materials of Chinese moths. In addition, molecular identification was performed based on the mitochondrial cytochrome c oxidase subunit I (*COI*) gene. The *COI* sequence of *C. andrei* showed the highest similarity to the available *Cricula* sequence in GenBank. Pairwise sequence comparison using MEGA version 11.0 (Mega Limited, Auckland, New Zealand) revealed 95.81% nucleotide identity between *C. andrei* and its closest congener, *C. trifenestrata*, providing additional molecular support for the morphological identification of the specimens. Total genomic DNA of a single adult specimen of *C. andrei* was extracted according to the instructions of the Aidlab Genomic DNA Extraction Kit (Aidlab Biotechnologies Co., Ltd., Beijing, China) and the quality of DNA was detected by 1% agarose gel electrophoresis (Sangon Biotech Co., Ltd., Shanghai, China). Thirteen pairs of specific primers were designed based on conserved regions of available Saturniidae mitochondrial genome sequences and synthesized at General Biosystems Co., Ltd. (Chuzhou, China) to amplify the mitochondrial genome sequence of *C. andrei* (Table 1). Primer design was performed using Primer Premier version 5.0 (Premier Biosoft International, Palo Alto, CA, USA), and primer parameters were optimized according to primer length, melting temperature, GC content, and specificity. The 13 primer pairs generated overlapping fragments covering the entire mitochondrial genome, with adjacent fragments sharing approximately 100–400 bp overlaps to ensure accurate sequence assembly. Mitochondrial DNA (mtDNA) fragments of *C. andrei* were amplified by polymerase chain reaction (PCR) using total genomic DNA as template. PCR was carried out in a 20 μL reaction system including 7 μL of sterile distilled water, 1 μL of extracted DNA template, 1 μL of forward primer, 1 μL of reverse primer, and 10 μL (1 unit) of Taq DNA polymerase (Takara Bio Inc., Dalian, China). PCR amplification was carried out at 95 °C for 5 min followed by 30 cycles of denaturation at 95 °C for 30 s, annealing at 50–55 °C for 1 min, and extension at 72 °C for 1–2 min, followed by extension at 72 °C for 10 min. PCR products were detected by 1% agarose gel electrophoresis, and the positive amplicons were purified using a DNA gel extraction kit (TransGen Biotech Co., Ltd., Beijing, China). The purified products were then sent to General Biosystems Co. for sequencing.

### 2.2. Sequence Assembly and Genome Annotation

The sequencing results from Section 2.1 were assembled based on PCR fragments obtained from Sanger sequencing. The forward and reverse sequencing chromatograms of each PCR fragment were manually inspected, and only high-confidence nucleotide calls were retained in the final mitochondrial genome sequence. DNAStar Lasergene version 7.1.0 (DNASTAR Inc., Madison, WI, USA) was used to assemble the complete mitochondrial genome according to the overlapping sequences of PCR fragments. Subsequently, the mitochondrial genomes of closely related species in GenBank were aligned by the NCBI BLAST online tool https://blast.ncbi.nlm.nih.gov/Blast.cgi (accessed on 5 July 2022) to verify sequence homology, confirm gene order, and validate the circular structure of the assembled mitogenome. Gene prediction and annotation were performed using the MITOS online server http://mitos.bioinf.uni-leipzig.de/index.py (Leipzig, Germany, accessed on 15 July 2022), parameters were set as follows: select invertebrate 5 code table (invertebrate mitochondrial genetic code is the standard translation table for insect mitochondrial genomes, including Lepidoptera), reference sequence lineage is RefSeq 89 Metazoa (it contains curated mitochondrial genome annotations for metazoan species), and the remaining parameters are the default values of MITOS [21]. The mitochondrial genome map was constructed using the Proksee online server https://proksee.ca/ (Vancouver, BC, Canada, accessed on 18 July 2022) [22]. Protein-coding sequences were translated into putative proteins according to the invertebrate mitochondrial genetic code using DNAStar Lasergene version 7.1.0 software. Both tRNAscan-SE version 2.0 search server http://lowelab.ucsc.edu/tRNAscan-SE/ (Santa Cruz, CA, USA, accessed on 15 July 2022) and MITOS server were used to identify the tRNA and rRNA genes [23]. MITOS for initial annotation, and tRNAscan for further verification. When discrepancies occurred between the two annotation tools, gene boundaries were manually inspected based on tRNA secondary structures, anticodon sequences, and sequence conservation with homologous mitochondrial genes from closely related Saturniidae species. The complete mitochondrial genome annotation file was prepared according to NCBI submission requirements and submitted to GenBank through the NCBI Submission Portal. The sequence has been publicly released in GenBank under accession number NC_084345 (It can be found at the following URL: https://www.ncbi.nlm.nih.gov/nuccore/NC_084345.1/ accessed on 5 January 2024). Overlapping regions between genes and intergenic spaces were manually counted. MEGA version 11.0 software was used to count the base content of A, T, C and G and relative synonymous codon usage (RSCU) in the mitochondrial genome [24]. For genes encoded on the minority strand, reverse-complement sequences were used before analysis. AT and GC skewness values were calculated according to the following formula: AT skewness = (A − T)/(A + T); GC skewness = (G − C)/(G + C) [15].

### 2.3. Phylogenetic Analysis

A total of 30 mitogenomes were downloaded from the GenBank database for phylogenetic reconstruction (Table 2). Focused primarily on species with complete mitochondrial genomes in Saturniidae and closely related Lepidoptera groups. The selected taxa were intended to represent major lineages of Lepidoptera and to maximize phylogenetic coverage based on currently available mitogenomic resources. *Macrostemum floridum* belongs to the order Trichoptera, which is the sister group of Lepidoptera within Amphiesmenoptera. Therefore, it was selected as an outgroup to root the phylogenetic tree. The nucleotide sequences of the 13 PCGs were aligned individually using MACSE version 2.1 (Montpellier, France) with the codon-aware alignment algorithm under the default parameters. Poorly aligned regions were subsequently removed using Gblocks version 0.91b (Barcelona, Spain) with relaxed settings, allowing smaller final blocks, gap positions within the final blocks, and less stringent flanking positions.

Phylogenetic trees were reconstructed using BI and ML methods [13,14]. The nucleotide sequences of 13 PCGs were concatenated into a combined dataset. The initial partition scheme was defined according to individual genes and codon positions. PartitionFinder2 version 2.1.1 (Edinburgh, UK) was used to determine the optimal partitioning scheme and nucleotide substitution models for BI analysis under the Bayesian information criterion (BIC), and the optimized partition scheme was subsequently applied in BI analyses [25]. BI analysis was performed in MrBayes version 3.2 (Tallahassee, FL, USA) using two independent runs [26]. Markov chains were run for one million generations, sampled every 100 generations, with the first 25% of sampled trees discarded as burn-in, and the remaining trees used to estimate posterior probabilities. Convergence between independent runs was assessed using the average standard deviation of split frequencies (ASDSF), with values below 0.01 indicating satisfactory convergence. IQ-TREE version 1.6.12 (Vienna, Austria) was used for ML analysis, and the built-in ModelFinder algorithm was applied to determine the best-fit nucleotide substitution models for each partition [27,28]. Node support was assessed using 1000 bootstrap replicates.

## 3. Results

### 3.1. Mitogenome Structure and Nucleotide Composition

**Figure 1:**
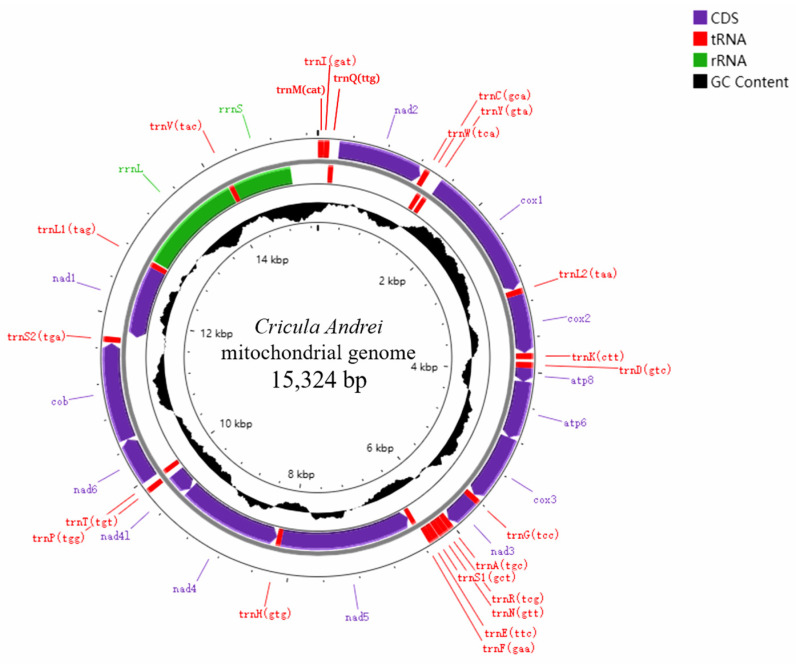
Map of the mitogenome of *Cricula andrei*.

The mitochondrial genome organization of *C. andrei* is shown in Figure 1. The mitogenome of *C. andrei* was 15,324 bp, including 13 PCGs, 22 tRNAs, 2 rRNAs and a control region (AT-rich region). The mitogenome was a double-stranded circular DNA consisting of the majority strand encoding 9 PCGs (*nad2*, *cox1*, *cox2*, *atp8*, *atp6*, *cox3*, *nad3*, *nad6* and *cob*) and 14 tRNAs (*trnM*, *trnI*, *trnW*, *trnL2*, *trnK*, *trnD*, *trnG*, *trnA*, *trnR*, *trnN*, *trnS1*, *trnE*, *trnT* and *trnS2*), and the minority strand encoding 4 PCGs (*nad1*, *nad4l*, *nad4* and *nad5*), 8 tRNAs (*trnV*, *trnL1*, *trnP*, *trnH*, *trnF*, *trnY*, *trnC* and *trnQ*) and 2 rRNAs (*rrnS* and *rrnL*). As shown in Table 3, the gene order and orientation are identical to those of the ancestral lepidopteran mitochondrial genome. The control region is located between *rrnS* and *trnM*, and several overlapping genes and short intergenic spacer regions are present throughout the genome.

The nucleotide composition, AT skewness and GC skewness of the *C. andrei* mitochondrial genome were analyzed and compared with those of other Lepidoptera species (Table 4). The nucleotide composition of the *C. andrei* mitogenome was biased toward A and T, with 78.12% of AT content (A: 39.0564%, T: 39. 0629%, C: 13.6975%, G: 8.1832%), and the content of AT in the control region was as high as 90.40%. Among the analyzed Lepidoptera species, the mitochondrial AT content ranged from 77.68% (*C. trifenestrata*) to 81.74% (*S. taiwanalis*), and the value observed in *C. andrei* was within this range. The mitogenome of *C. andrei* had an AT-skew of −0.0001 and a GC-skew of −0.25, similar to most examined Lepidoptera species, which showed AT-skew values ranging from −0.04 to 0.01 and consistently negative GC-skew values. These results indicated that *C. andrei* exhibited an approximately balanced distribution of nucleotide A and T but a higher proportion of nucleotide C than G. The negative GC-skew observed in *C. andrei* and other Lepidoptera mitochondrial genomes indicates a conserved nucleotide compositional asymmetry [5].

### 3.2. Protein-Coding Genes and Codon Usage

**Figure 2:**
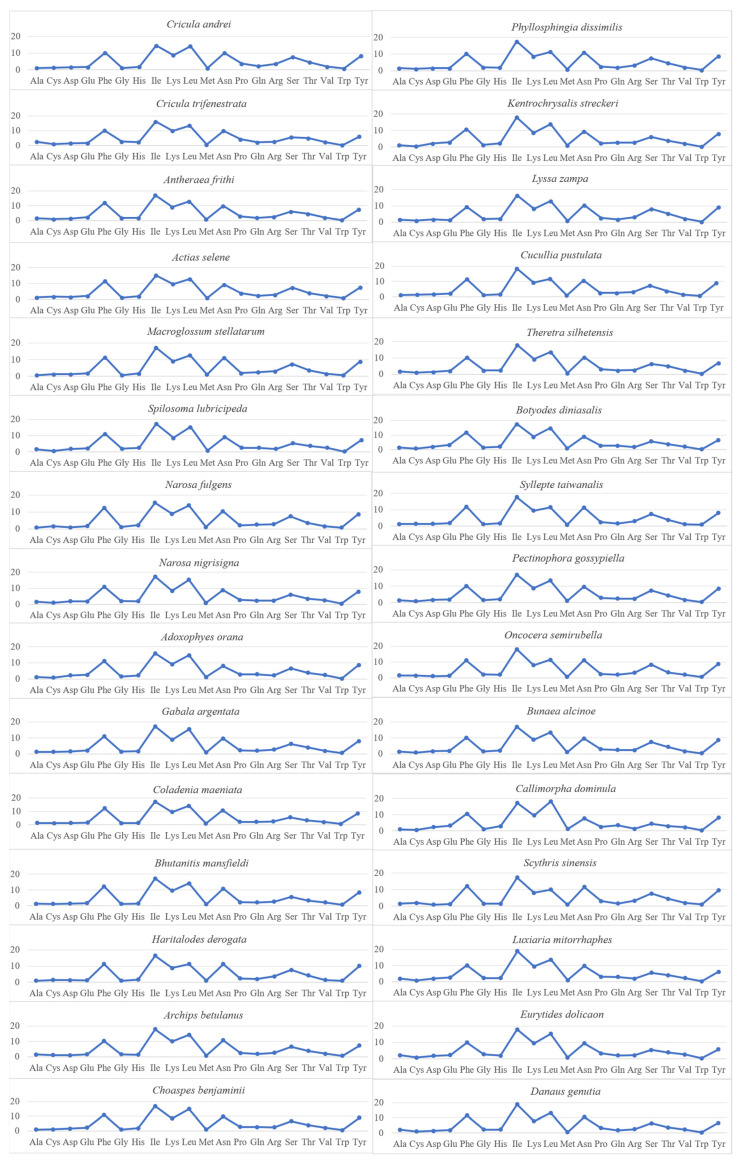
Codon distribution patterns in various Lepidoptera species. The y-coordinate is the proportion of codons per 100 codons.

**Figure 3:**
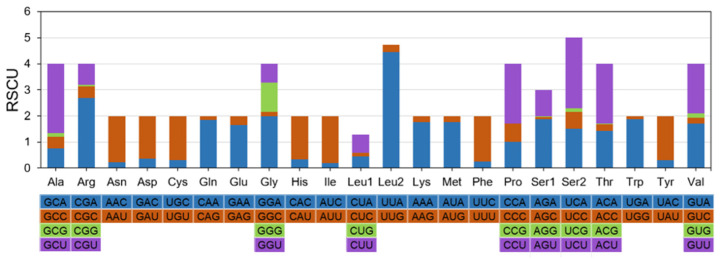
The Relative Synonymous Codon Usage (RSCU) of the mitochondrial genome of *Cricula andrei*. Codon families are plotted on the X axis.

The total length of 13 PCGs was 11,221 bp in the mitochondrial genome of *C. andrei* (Table 3). Codon usage analysis showed that 12 out of 13 PCGs had a start codon ATN (ATG, ATT, ATC), while for *cox 1* it was annotated to start with CGA, and 11 out of 13 PCGs had a stop codon TAA, while for *cox 2* it was putative T and *nad 3* which was putative TAG (Table 3). Amino acid usage frequency (AAUF) analysis showed that Ile, Leu, Phe and Asn were the most frequently used amino acids in *C. andrei*, and Trp was the least frequently used amino acid, which was similar to the AAUF of PCGs in other 30 species of Lepidoptera (Figure 2). Relative synonymous codon usage (RSCU) analysis showed that the codons used more frequently in *C. andrei* were UUA, CGA, AUU, UUU and AUA, while the codons used less frequently were CUG, CCG and AGG (Figure 3). Previous studies have shown that UUA (Leu) is typically the most frequently used codon in lepidopteran mitochondrial genomes, whereas codons ending with G or C are generally underrepresented [5]. The codon usage pattern of *C. andrei* is highly consistent with that reported for other Lepidoptera. Most preferred codons ended with A or U, indicating a strong AU bias in synonymous codon usage.

### 3.3. Transfer RNA (tRNA) and Ribosomal RNA (rRNA) Genes

**Figure 4:**
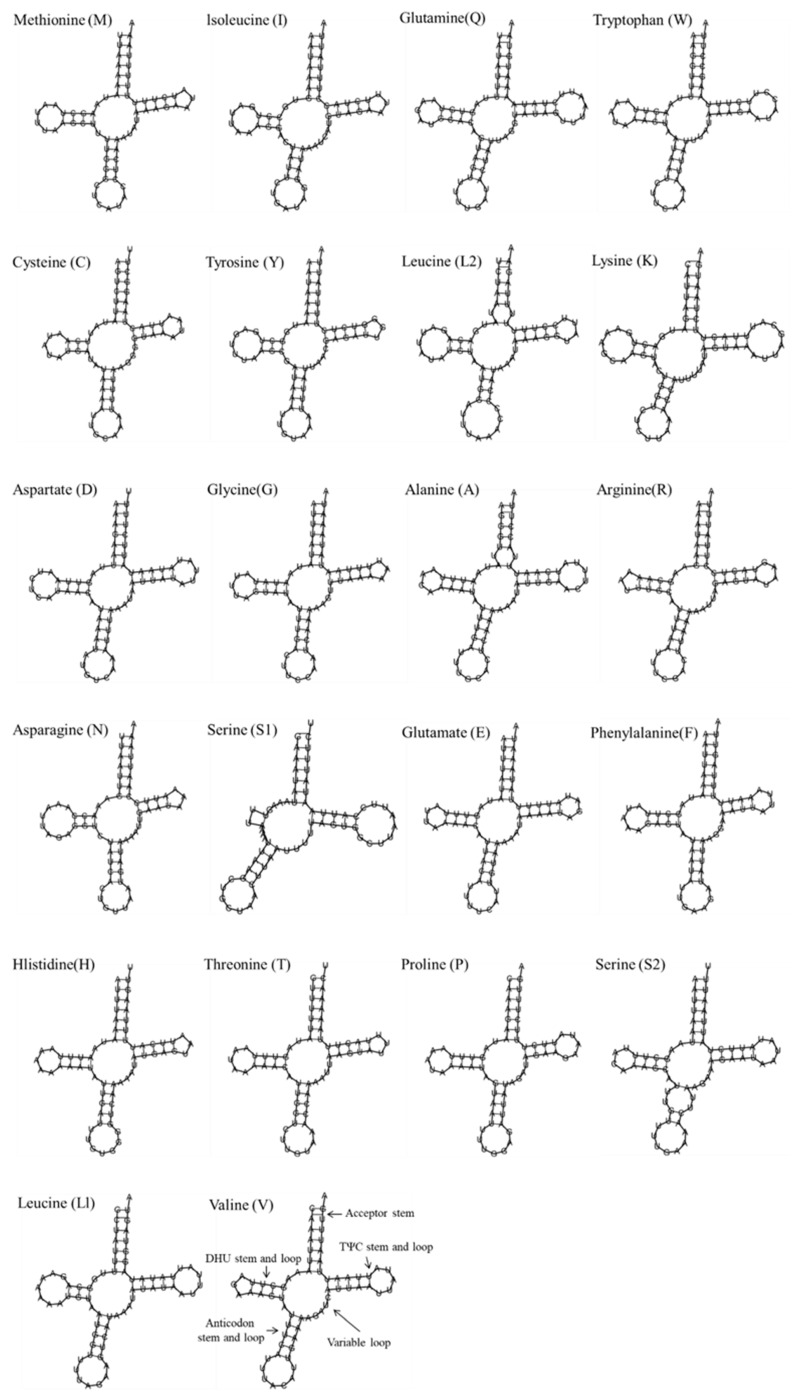
Putative secondary structures of the 22 tRNA genes of the *Cricula andrei* mitogenome. Structural elements of tRNAs are labeled in the representative structure of *trnV*, including the acceptor stem, DHU stem and loop, anticodon stem and loop, TΨC stem and loop, and variable loop.

There were 22 tRNAs in the mitochondrial genome of *C. andrei*, 21 of which had the typical cloverleaf structures, while the tRNAS1 exhibited a reduced D-loop structure (Figure 4). The length of tRNAs ranged from 64 bp (*trnC* and *trnF*) to 71 bp (*trnK*). A total of 27 mismatched base pairs were identified in the stem regions of the 22 tRNAs, including 26 GU base pairs and 1 UU base pair, and these tRNAs were *trnM*, *trnI*, *trnQ*, *trnW*, *trnC*, *trnY*, *trnG*, *trnA*, *trnS1*, *trnF*, *trnH*, *trnP*, *trnL1* and *trnV* (Table 5). The remaining eight tRNAs (*trnL2*, *trnK*, *trnD*, *trnR*, *trnN*, *trnE*, *trnT* and *trnS2*) showed complete Watson–Crick base pairing without mismatches. The gene rearrangement phenomenon of tRNAs occurs frequently among the *trnM*, *trnI* and *trnQ* genes in lepidopteran mitochondrial genome, and the general arrangement is *trnM*-*trnI*-*trnQ* or *trnI*-*trnQ*-*trnM*. Here, the arrangement of *C. andrei* was *trnM*-*trnI*-*trnQ*, which was different from the ancestral insects’ order *trnI*-*trnQ*-*trnM* [3].

There were two rRNAs (*rrnL* and *rrnS*) in the mitochondrial genome of *C. andrei*. The *rrnL* gene (16S rRNA) was 1352 bp in length and was located between *trnL1* and *trnV*, whereas the *rrnS* gene (12S rRNA) was 765 bp in length and was located between *trnV* and the control region. In Lepidoptera, the *rrnL* and *rrnS* genes generally show conserved positions, with average lengths of approximately 1353 bp and 774 bp, respectively [5]. The lengths of both *rrnL* and *rrnS* genes in *C. andrei* were comparable to the reported averages, indicating that their organization and size are consistent with the mitochondrial rRNA characteristics commonly observed in Lepidoptera. The AT content of two rRNAs was 82.94% (Table 4). The high AT bias observed in rRNA genes is consistent with the nucleotide composition characteristics commonly reported in lepidopteran mitochondrial genomes, where AT contents are typically higher than 80% (Table 4).

### 3.4. The Control Region (AT-Rich Region)

**Figure 5:**
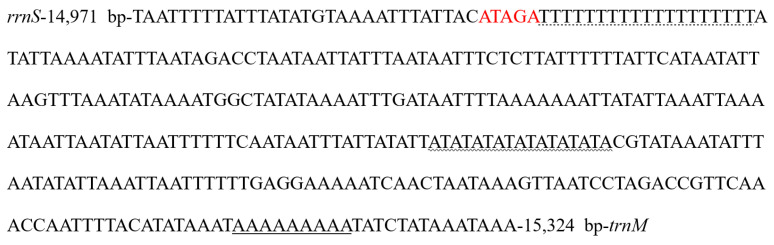
The control region of *Cricula andrei*. The ATAGA motif is marked red. The poly-T is marked with a dotted line. The microsatellite TA repeats is indicated by wavy lines and the poly-A is underlined.

Figure 5 showed the control region (CR) of mitochondrial genome of *C. andrei*. It was located between *rrnS* and *trnM*, with a size of 354 bp, and the AT content of this region was the highest in the whole mtDNA sequence, reaching 90.40%. The length of mitochondrial CRs varies considerably among Lepidoptera species, even in different geographic populations. Based on the CR regions of the 706 lepidopteran species that have been statistically analyzed, the average length of the CR regions is around 400 bp. Although the CR of *C. andrei* is relatively short, it still falls within the common range for lepidopterans [5]. An ATAGA motif was identified in the CR at positions 31–35 bp, followed immediately by a 19 bp poly-T stretch (positions 36–54 bp). A microsatellite region composed of tandem (AT)n repeats was detected at approximately positions 207–222 bp, containing eight AT repeat units (AT)_8_. In addition, a 9 bp poly-A tract was identified at positions 318–326 bp. Several commonly observed elements have been frequently identified in the CRs of lepidopteran mitochondrial genomes, including the ATAGA motif, a 15–20 bp poly-T stretch, and a poly-A tract. However, the occurrence of these motifs is not universal across all lepidopteran lineages. Species within Hepialoidea lack the “ATAGA” motif and poly-A tract in their mitochondrial CR, but retain only the poly-T structure, which is located at the beginning of the CR and followed by the 5′ end of 12S rRNA [5].

### 3.5. Intergenic Spacers and Overlap Region

A total of 20 intergenic spaces were identified in the mitochondrial genome of *C. andrei*, with a total length of 203 bp (Table 3). The length of these spacers ranged from 1 to 48 bp. The shortest spacers (1 bp) were detected between *trnL2* and *cox2*, *trnR* and *trnN*, and *nad1* and *trnL1*, whereas the longest spacer (48 bp) occurred between *trnQ* and *nad2*.

**Figure 6:**
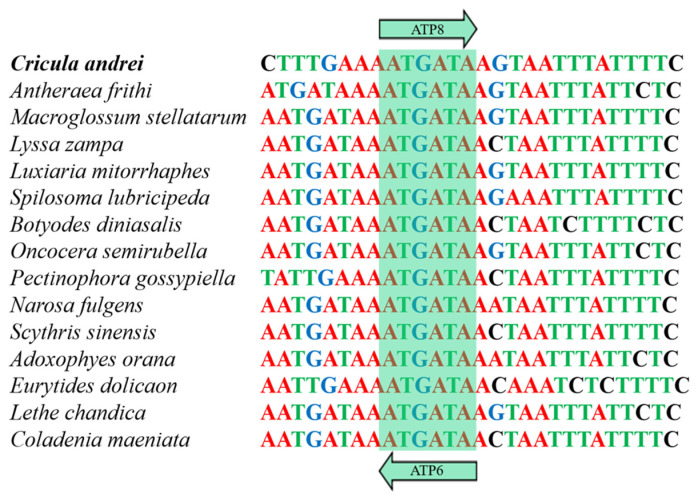
Alignment of overlapping region between *atp8* and *atp6*. The nucleotide letters are color-coded for clarity: adenine (A), thymine (T), cytosine (C), and guanine (G) are shown in red, green, black, and blue, respectively.

The number of gene-overlapping regions was less than that of intergenic spacer regions in the mitochondrial genome of *C. andrei* (Table 3). Only 8 overlapping regions were found, and the total number of nucleotides in this region was 25 bp. The length of these overlaps ranged from 1 to 8 bp. The shortest overlaps (1 bp) were detected between *atp6* and *cox3*, *trnH* and *nad4*, and *nad6* and *cob*, respectively. The longest overlaps (8 bp) occurred between *trnW* and *trnC*. The remaining intergenic spacers and overlaps were distributed among different gene boundaries, and their detailed locations and lengths are provided in Table 3. The overlapping region between *atp8* and *atp6* was compared with the corresponding regions from 15 Lepidoptera species representing 10 families to assess the conservation of this gene boundary (Figure 6). The 3′ end of *atp8* and the 5′ end of *atp6* in the *C. andrei* mitogenome shared a 7 bp overlapping sequence (“ATGATAA”), which was also present in the analyzed Lepidoptera species.

### 3.6. Phylogenetic Analyses

**Figure 7:**
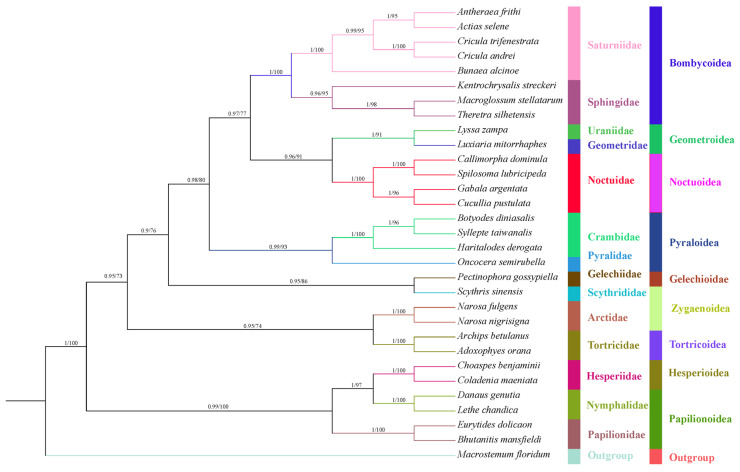
Phylogenetic tree reconstructed using Bayesian inference (BI) and Maximum likelihood (ML) methods based on the protein-coding genes. *Macrostemum floridum* was used as the outgroup. Node values are shown as Bayesian posterior probabilities (BPP) and ML bootstrap support (BS), respectively. Colored bars indicate family (**left**) and superfamily (**right**) assignments of the sampled taxa.

The mitochondrial genomes of 30 species of lepidopterans belonging to 9 superfamilies and 14 families were obtained from GenBank (*Macrostemum floridum* was regarded as outgroups). Based on the sequences of 13 PCGs of the mitochondria, the phylogenetic trees of Lepidoptera species were constructed using BI and ML methods. The BI and ML analyses generated highly congruent topologies. Therefore, a single phylogenetic tree was presented, with both ML bootstrap support values (BS) and Bayesian posterior probabilities (PP) shown at the nodes (Figure 7). Most internal nodes received strong support (BS ≥ 90 and PP ≥ 0.95), indicating strong support for the inferred phylogenetic relationships.

The constructed phylogeny recovered the major lepidopteran lineages with clear relationships among families and superfamilies. Within the superfamily Bombycoidea, Saturniidae and Sphingidae formed two well-supported evolutionary lineages. The family Saturniidae was recovered as a strongly supported clade, in which *C. andrei* clustered together with *C. trifenestrata*, representing the monophyletic genus *Cricula* (BS = 100; BPP = 1.00). The phylogenetic relationship within Saturniidae was therefore reconstructed as (*Actias selene* + *Antheraea frithi*) + (*C. andrei* + *C. trifenestrata*). In addition, the family Sphingidae formed a distinct and strongly supported clade, where *Kentrochrysalis streckeri* and *Macroglossum stellatarum* were recovered as sister species and together formed a sister group relationship with *Theretra silhetensis*. These results indicated a close but independent evolutionary relationship among the major bombycoid lineages.

The remaining lepidopteran superfamilies showed distinct family-level clustering. Within Geometroidea, *Luxiaria mitorrhaphes* (Geometridae) and *Lyssa zampa* (Uraniidae) represented separate lineages. The sampled Noctuoidea taxa were divided into two major lineages, with *Callimorpha dominula* and *Spilosoma lubricipeda* forming a supported clade, while *Cucullia pustulata* and *Gabala argentata* clustered together. The superfamily Pyraloidea showed clear separation between Crambidae and Pyralidae. For the butterfly lineage Papilionoidea, the sampled families Hesperiidae, Nymphalidae, and Papilionidae were recovered as distinct but closely related groups. *Choaspes benjaminii* and *Coladenia maeniata* formed a strongly supported Hesperiidae lineage, while *Danaus genutia* and *Lethe chandica* represented the Nymphalidae lineage. The two Papilionidae species, *Bhutanitis mansfieldi* and *Eurytides dolicaon*, clustered together, supporting the monophyly of the Papilionidae taxa.

The mitochondrial PCGs dataset provided strong resolution for relationships among most sampled lepidopteran groups and confirmed the phylogenetic placement of *C. andrei* within Saturniidae.

## 4. Discussion

Mitochondrial genome sequences of lepidopterans are relatively conservative, mostly between 15 and 16 kb in size, and a few species have less than 15 kb or more than 16 kb due to lack of CR or long CR, such as the *Glyphodes pyloalis* (14,960 bp), *Danaus gilippus* (14,869 bp), *Meandrusa sciron* (14,875 bp), *Ectropis obliqua* (16,535 bp), *Papilio maraho* (16,094 bp), *Pergesa acteus* (16,035 bp) and *Dierna strigata* (16,087 bp). In this study, the full length of mitochondrial genome of *C. andrei* was 15,324 bp, which contained 13 PCGs, 22 tRNAs, 2 rRNAs and a non-coding AT-rich region [2,3,5].

Although ATN codons are considered the primary initiation signals in insect mitochondrial genomes, variations in start codon usage have been reported among Lepidoptera [1,29]. Previous studies have demonstrated that *cox1* exhibits relatively high variability in its initiation region compared with other mitochondrial PCGs [16]. The putative CGA start codon has been reported in several lepidopteran mitochondrial genomes, although its functional role as a translation initiation codon remains uncertain [3,5,30]. Among the 13 PCGs of the *C. andrei* mitochondrial genome, except for *cox1*, which was annotated with CGA as the putative start codon, the remaining PCGs started with ATN codons, which represent the predominant initiation pattern in lepidopteran mitochondrial genomes [5,15]. Four types of mitochondrial stop codons have been discovered in Lepidoptera, among which TAA is the most common, followed by TAG, TA and T [18,19]. Such incomplete stop codons are believed to be able to form complete TAA through transcriptional polyadenylation during maturation [3,5].

UUA (Leu) is the most frequently used codon in *C. andrei* and other lepidopteran mitochondrial genomes, and most preferred codons ended with A or U at the third codon position [5,19]. Such A/U-ending codon preference represents a common evolutionary feature of insect mitochondrial genomes [2,5,15,16]. The relationship between nucleotide composition and codon usage bias may mainly result from asymmetric mutational pressure during mitochondrial genome evolution. The enrichment of AT nucleotides may contribute to the preferential usage of A/U-ending codons, whereas G/C-ending synonymous codons tend to be used less frequently [2,5,16].

Of the 22 tRNAs of *C. andrei* mitochondria, 21 were typical cloverleaf structures, but the tRNAS1 lacked a D-loop. The absence of D-loop is common in the mitochondrial genomes of Lepidoptera, and it may indicate that the *trnS1* is early evolved in metazoans [31,32]. There were 27 base mispairings in the mitochondrial tRNA of *C. andrei*, which also exists in most lepidopterans. However, it does not affect the correct translation of the genetic code, because the existence of wobble bases can adjust the secondary structure of RNA [33]. Like most insects, the two rRNAs (16S rRNA and 12S rRNA) in the mitochondria of *C. andrei* were located between *trnL1* and *trnV*, and *trnV* and control region, respectively.

Control region (CR), also known as D-loop region, is related to the initiation and transcription of gene replication [5,13]. Due to its high AT content, it is also called the AT-rich region. By comparison, it is found that differences in mitochondrial genome length among lepidopterans are largely associated with variation in CR length [5,6,7]. The CR of *C. andrei* was 354 bp in length, and its AT content was as high as 90.40%. Like most lepidopterans, this region was located between 12S rRNA and *trnM*, and contained several commonly observed structural elements, such as “ATAGA”motif, poly-T and poly-A structures and microsatellite (AT)n element [15,16,19,34]. These typical structural elements are considered characteristic features of lepidopteran mitochondrial control regions, although the length variation in the control region is mainly associated with differences in tandem repeat expansion [5,6,7,34]. However, the composition and organization of conserved elements within the CR may vary among different Lepidoptera lineages [34]. For example, species of Hepialoidea lack the typical ATAGA motif and poly-A structure, indicating that some conserved elements of the mitochondrial CR can undergo structural divergence during evolution [16,29,34]. Such variation suggests that although the CR plays conserved roles in mitochondrial replication and transcription, its sequence composition and structural organization may evolve under different constraints among lepidopteran lineages [2,3,34].

The mitochondrial genome of Lepidoptera insects has a very compact gene arrangement structure and short non-coding sequences, which makes gene rearrangement, inversion, and translocation rare during mutation processes [3]. Two major types of mitochondrial gene orders have been reported in Lepidoptera, occurring between the *trnM*, *trnI*, and *trnQ* genes. The first type is found in the mitochondria of most lepidopterans and is characterized by the *trnM*-*trnI*-*trnQ* arrangement, which is consistent with the first sequenced lepidopteran insect, *Bombyx mori*, and the other order is *trnI*-*trnQ*-*trnM*, which is mostly found in the primitive groups of Lepidoptera, and is consistent with the mitochondrial genome of *Drosophila* [29,35,36]. The sequence of *trnM*-*trnI*-*trnQ* in *C. andrei* was consistent with that of *Bombyx mori* and most lepidopterans.

There are also some conserved gene-overlapping regions in Lepidoptera, such as a 7 bp “ATGATAA” between *atp6* and *atp8*, which is the same as that of *C. andrei*, but in Micropteroidea, the overlapping region of *atp6* and *atp8* is “ATGATAG” [5]. In Hymenoptera there are even five different overlapping regions between *atp6* and *atp8* [37,38].

Molecular phylogenetic analysis based on the mitochondrial genome of insects provides a lot of reference information for the study of its classification and genetics [5,6,9,36]. To date, molecular phylogenetic studies specifically focusing on *C. andrei* remain unavailable. Here, 30 species of Lepidoptera containing 14 families were used to construct a phylogenetic tree based on 13 PCGs sequences of mitogenomes using BI and ML methods. The high concordance between BI and ML analyses and the strong support values at most nodes demonstrate the reliability of mitochondrial PCG sequences for reconstructing lepidopteran phylogenetic relationships [5,6,36]. *C. andrei* was consistently grouped with *C. trifenestrata*, forming a well-supported monophyletic lineage of the genus *Cricula*. This result is consistent with their current taxonomic classification and indicates that the two *Cricula* species share a recent common ancestor [30]. Furthermore, the position of *C. andrei* within Saturniidae was consistent with previous lepidopteran phylogenetic studies, where Saturniidae generally forms a well-supported lineage within Bombycoidea [30,39,40]. The close relationship among *Cricula*, *Actias*, and *Antheraea* suggests their relatively recent common ancestry within Saturniidae and supports the close evolutionary affinity among these genera. The separation of Saturniidae and Sphingidae into distinct, well-supported lineages in the phylogenetic tree indicates that these two families represent separate evolutionary lineages within Bombycoidea. Although both families possess several similar biological characteristics, their mitochondrial evolutionary histories support their status as distinct lineages. In Gelechioidea, Gelechiidae and Scythrididae were recovered as sister lineages with moderate-to-high support (BPP = 0.95; BS = 86). However, the phylogenetic relationships among gelechioid families remain controversial due to incomplete taxon sampling and complex evolutionary histories [41,42]. Therefore, broader taxon sampling and nuclear genomic data are required to further resolve relationships within this superfamily. The relationships among Papilionoidea families were generally consistent with previous studies, with Hesperiidae, Nymphalidae, and Papilionidae forming independent lineages [40]. The monophyly of each sampled family was strongly supported, indicating that mitochondrial genome data provided sufficient phylogenetic signals for resolving relationships among the sampled butterfly families.

Nevertheless, the current data on lepidopteran mitochondria are limited and unevenly distributed among taxa. Current sampling is still insufficient to fully resolve phylogenetic relationships among different Lepidoptera lineages. In this study, the complete mitochondrial genome of *C. andrei* was characterized and incorporated into phylogenetic analyses, providing additional molecular resources for Saturniidae. However, the present study was based primarily on mitochondrial genomic information and included a limited number of representative taxa; therefore, further studies incorporating broader taxonomic sampling, particularly for underrepresented lineages, are needed. Future studies integrating mitochondrial genomic data with nuclear genomic datasets will help to resolve remaining uncertainties in the evolutionary relationships of Lepidoptera and provide deeper insights into the diversification history of this group.

## 5. Conclusions

The complete mitochondrial genome sequence of *C. andrei* was analyzed and characterized, and a series of analyses on its genome structure, base composition, PCGs, codon usage, RNA genes, overlapping and intergenic spacer regions, CR and phylogenetic accounts were conducted. This work enriches the mitochondrial genome database of Lepidoptera. However, there is a huge number of Lepidoptera species, and more mitochondrial genome information needs to be analyzed in the future to improve the database, so as to provide more basis and reference for its classification and evolution.

## Figures and Tables

**Table 1:** Primers used for polymerase chain reaction amplification.

Primer Names	Primer Sequence (5′→3′)	Tm (°C)
F1	ATAAAGTGCCTGATTAAAGGA	56
R1	TATTAACGAGATTGGGTCTC	54
F2	AAACTAATAATCTTCAAAATTAT	56
R2	AAAATAATTTGTTCTATTAAAG	56
F3	CCAGTTTTAGCTGGAGCTATTAC	62
R3	GTAAATGCTAATTTACTATAAAATGG	62
F4	ATTTGTGGAGCTAATCATAG	56
R4	GGTCAGGGACTATAATCTAC	58
F5	CATATAATCCCTCAAGGAACTCC	64
R5	TAAATTATAACCCTAATTTGTTCA	58
F6	TTGATGTAGAAATTGCCTTA	52
R6	GATTTAATTTTAGAAATAGTAAG	56
F7	ATAATATACAGTCAAGCCAGTAG	60
R7	GATTTGTGGGGTCAAAAATATGA	66
F8	AATAGATATAACTACTCTTACAG	56
R8	TATTAAGAATTTGAATTTGTGG	56
F9	GTAGGAATTAATCTACCCTC	54
R9	CCTCCTCAAATTCAGTTTAC	60
F10	TTAATTGATTTACCAACTCCCAC	64
R10	TGAGTTCGAGGTACATTGCC	60
F11	GCATTTGTTTTGAAAACTTAAG	58
R11	CATAAATAAATGAATTTATGATCC	60
F12	GTTTGAACTCAGATCATGTAAG	60
R12	TTAATTGAAAGTGGATTTGATTG	60
F13	ACACTTTCCAGTACCTCTAC	56
R13	TTAGTGTAAGATGCACAGTAG	60

**Table 2:** Information on mitochondrial genome data for phylogenetic tree construction.

Superfamily	Family	Species	Size (bp)	Accession Number
Bombycoidea	Saturniidae	*Cricula andrei*	15,324	NC_084345
		*Cricula trifenestrata*	15,425	KY644697
		*Antheraea frithi*	15,338	NC_027071
		*Bunaea alcinoe*	15,305	NC_061295
		*Actias selene*	15,236	NC_018133
	Sphingidae	*Kentrochrysalis streckeri*	15,253	MZ593600
		*Theretra silhetensis*	15,377	NC_082872
		*Macroglossum stellatarum*	15,290	NC_037441
Geometroidea	Uraniidae	*Lyssa zampa*	15,571	MZ713634
	Geometridae	*Luxiaria mitorrhaphes*	15,340	NC_061239
Noctuoidea	Noctuidae	*Spilosoma lubricipeda*	15,369	MK903030
		*Cucullia pustulata*	15,378	NC_062181
		*Gabala argentata*	15,337	NC_026842
		*Callimorpha dominula*	15,496	NC_027094
Pyraloidea	Crambidae	*Botyodes diniasalis*	15,219	NC_073002
		*Syllepte taiwanalis*	15,264	NC_061245
		*Haritalodes derogata*	15,258	OQ472986
	Pyralidae	*Oncocera semirubella*	15,290	MT012820
Gelechioidea	Gelechiidae	*Pectinophora gossypiella*	15,202	NC_065403
Zygaenoidea	Limacodidae	*Narosa fulgens*	15,296	NC_072151
		*Narosa nigrisigna*	15,292	NC_041304
	Scythrididae	*Scythris sinensis*	15,216	NC_048471
Tortricoidea	Tortricidae	*Adoxophyes orana*	15,343	NC_021396
		*Archips betulanus*	16,089	MW924658
Papilionoidea	Papilionidae	*Eurytides dolicaon*	15,354	LR031513
		*Bhutanitis mansfieldi*	14,994	NC_037863
	Nymphalidae	*Lethe chandica*	15,249	NC_070054
		*Danaus genutia*	15,255	NC_065309
Hesperioidea	Hesperiidae	*Coladenia maeniata*	15,284	NC_079683
		*Choaspes benjaminii*	15,272	JX101620

**Table 3:** Sequence characteristics of the mitochondrial genome of *Cricula andrei*.

Gene	Start	Stop	Strand	Anticodon	Length	Ovl/nc	Start and Stop Codons
*trnM*	1	66	+	CAT	66	3	/
*trnI*	70	134	+	GAT	65	−3	/
*trnQ*	132	200	−	TTG	69	48	/
*nad2*	249	1262	+	/	1014	8	ATT/TAA
*trnW*	1271	1338	+	TCA	68	−8	/
*trnC*	1331	1394	−	GCA	64	25	/
*trnY*	1420	1484	−	GTA	65	11	/
*cox1*	1496	3028	+	/	1533	3	CGA/TAA
*trnL2*	3032	3097	+	TAA	66	1	/
*cox2*	3099	3780	+	/	682	0	ATG/T
*trnK*	3781	3851	+	CTT	71	32	/
*trnD*	3884	3951	+	GTC	68	0	/
*atp8*	3952	4119	+	/	168	−7	ATC/TAA
*atp6*	4113	4790	+	/	678	−1	ATG/TAA
*cox3*	4790	5578	+	/	789	2	ATG/TAA
*trnG*	5581	5645	+	TCC	65	0	/
*nad3*	5646	5999	+	/	354	−2	ATT/TAG
*trnA*	5998	6064	+	TGC	67	4	/
*trnR*	6069	6133	+	TCG	65	1	/
*trnN*	6135	6199	+	GTT	65	7	/
*trnS1*	6207	6272	+	GCT	66	0	/
*trnE*	6273	6337	+	TTC	65	3	/
*trnF*	6341	6404	−	GAA	64	−2	/
*nad5*	6403	8148	−	/	1746	0	ATT/TAA
*trnH*	8149	8213	−	GTG	65	−1	/
*nad4*	8213	9553	−	/	1341	0	ATG/TAA
*nad4l*	9554	9844	−	/	291	3	ATG/TAA
*trnT*	9848	9912	+	TGT	65	0	/
*trnP*	9913	9977	−	TGG	65	2	/
*nad6*	9980	10,513	+	/	534	−1	ATT/TAA
*cob*	10,513	11,664	+	/	1152	6	ATG/TAA
*trnS2*	11,671	11,736	+	TGA	66	18	/
*nad1*	11,755	12,693	−	/	939	1	ATG/TAA
*trnL1*	12,695	12,762	−	TAG	68	17	/
*rrnL*	12,780	14,131	−	/	1352	8	/
*trnV*	14,140	14,205	−	TAC	66	0	/
*rrnS*	14,206	14,970	−	/	765	/	/
Control Region	14,971	15,324	/	/	354	/	/

Note: Ovl/nc represents the overlaps or intergenic spacers between adjacent genes. Negative values indicate overlapping regions, while positive values indicate the length of intergenic spacers.

**Table 4:** Comparison of base composition and skewness of mitochondrial genomes between *Cricula andrei* and other Lepidoptera.

Species	Size (bp)	A%	G%	T%	C%	A + T%	ATskewness	GCskewness
Mitochondrial genome								
*C. andrei*	15,324	39.0564	8.18	39.0629	13.70	78.12	−0.0001	−0.25
*C. trifenestrata*	15,425	39.27	8.07	38.41	14.24	77.68	0.01	−0.28
*A. frithi*	15,338	39.39	7.69	40.81	12.11	80.20	−0.02	−0.22
*B. diniasalis*	15,219	39.55	7.84	41.28	11.33	80.83	−0.02	−0.18
*S. taiwanalis*	15,264	40.52	7.47	41.22	10.79	81.74	−0.01	−0.18
*O. semirubella*	15,290	39.68	7.60	41.66	11.06	81.34	−0.02	−0.19
*S. lubricipeda*	15,369	40.05	7.37	41.33	11.26	81.38	−0.02	−0.21
*N. fulgens*	15,296	39.16	7.79	41.15	11.91	80.31	−0.02	−0.21
*N. nigrisigna*	15,292	38.97	7.67	42.24	11.12	81.21	−0.04	−0.18
*G. argentata*	15,337	39.64	7.56	42.05	10.75	81.69	−0.03	−0.17
*C. dominula*	15,496	40.07	7.58	40.95	11.40	81.02	−0.01	−0.20
*S. sinensis*	15,216	38.70	7.74	42.15	11.42	80.85	−0.04	−0.19
*L. mitorrhaphes*	15,340	41.05	7.71	40.03	11.22	81.08	0.01	−0.19
*E. dolicaon*	15,354	40.20	8.07	39.64	11.97	79.84	0.01	−0.19
*D. genutia*	15,255	38.96	7.49	42.27	11.28	81.23	−0.04	−0.20
*B. mansfieldi*	14,994	40.10	7.26	41.44	11.19	81.54	−0.02	−0.21
*C. benjaminii*	15,272	40.08	7.52	40.70	11.70	80.78	−0.01	−0.22
*L. chandica*	15,249	38.40	7.73	41.42	12.45	79.82	−0.04	−0.23
PCG								
*C. andrei*	11,221	31.87	11.58	44.53	12.02	76.40	−0.17	−0.02
*C. trifenestrata*	11,219	31.54	11.62	44.05	12.78	75.59	−0.17	−0.05
*A. frithi*	11,208	33.14	10.82	45.56	10.48	78.70	−0.16	0.02
*B. diniasalis*	11,205	33.82	10.70	45.57	9.92	79.39	−0.15	0.04
*S. taiwanalis*	11,193	34.48	10.22	45.90	9.40	80.38	−0.14	0.04
*O. semirubella*	11,181	33.92	10.39	46.02	9.67	79.94	−0.15	0.04
*S. lubricipeda*	11,208	34.37	10.28	45.62	9.73	79.99	−0.14	0.03
*N. fulgens*	11,163	32.63	11.05	45.78	10.53	78.41	−0.17	0.02
*N. nigrisigna*	11,172	33.44	10.54	46.07	9.94	79.51	−0.16	0.03
*G. argentata*	11,203	33.92	10.19	46.41	9.48	80.33	−0.16	0.04
*C. dominula*	11,215	34.25	10.36	45.96	9.43	80.21	−0.15	0.05
*S. sinensis*	11,187	33.59	10.49	45.99	9.92	79.58	−0.16	0.03
*L. mitorrhaphes*	11,211	34.12	10.65	45.44	9.79	79.56	−0.14	0.04
*E. dolicaon*	11,193	33.41	11.17	44.81	10.60	78.23	−0.15	0.03
*D. genutia*	11,176	33.91	10.42	45.89	9.78	79.80	−0.15	0.03
*B. mansfieldi*	11,189	34.70	9.55	46.01	9.71	80.71	−0.14	−0.01
*C. benjaminii*	11,153	33.17	10.54	46.01	10.28	79.18	−0.16	0.01
*L. chandica*	11,194	32.59	10.98	45.61	10.82	78.20	−0.17	0.01
tRNA								
*C. andrei*	1454	41.20	11.28	39.06	8.46	80.26	0.03	0.14
*C. trifenestrata*	1453	41.43	11.36	39.02	8.19	80.45	0.03	0.16
*A. frithi*	1476	42.28	10.77	39.50	7.45	81.78	0.03	0.18
*B. diniasalis*	1476	41.33	10.57	40.58	7.52	81.91	0.01	0.17
*S. taiwanalis*	1481	42.88	10.20	39.64	7.29	82.52	0.04	0.17
*O. semirubella*	1480	41.82	10.81	39.93	7.43	81.75	0.02	0.19
*S. lubricipeda*	1463	41.49	10.73	40.19	7.59	81.68	0.02	0.17
*N. fulgens*	1458	42.25	10.56	39.85	7.34	82.10	0.03	0.18
*N. nigrisigna*	1487	42.84	10.29	39.41	7.46	82.25	0.04	0.16
*G. argentata*	1472	41.24	11.07	39.67	8.02	80.91	0.02	0.16
*C. dominula*	1469	41.66	10.76	39.89	7.69	81.55	0.02	0.17
*S. sinensis*	1471	41.26	10.74	40.24	7.75	81.50	0.01	0.16
*L. mitorrhaphes*	1471	42.01	10.40	39.90	7.68	81.91	0.03	0.15
*E. dolicaon*	1388	42.07	11.02	38.98	7.93	81.05	0.04	0.16
*D. genutia*	1445	41.38	10.66	40.00	7.96	81.38	0.02	0.15
*B. mansfieldi*	1465	42.39	10.58	39.66	7.37	82.05	0.03	0.18
*C. benjaminii*	1467	41.99	10.70	39.40	7.91	81.39	0.03	0.15
*L. chandica*	1452	41.12	11.43	39.88	7.58	81.00	0.02	0.20
rRNA								
*C. andrei*	2117	42.51	11.67	40.43	5.39	82.94	0.03	0.37
*C. trifenestrata*	2161	42.57	11.29	41.00	5.14	83.57	0.02	0.37
*A. frithi*	2157	43.86	10.71	40.33	5.10	84.19	0.04	0.35
*B. diniasalis*	2124	43.64	9.98	41.34	5.04	84.98	0.03	0.33
*S. taiwanalis*	2156	42.90	9.83	42.39	4.87	85.29	0.01	0.34
*O. semirubella*	2111	44.77	9.95	40.45	4.83	85.22	0.05	0.35
*S. lubricipeda*	2174	42.87	9.94	42.18	5.01	85.05	0.01	0.33
*N. fulgens*	2067	44.12	10.01	40.93	4.93	85.05	0.04	0.34
*N. nigrisigna*	2134	44.61	9.28	41.28	4.83	85.89	0.04	0.32
*G. argentata*	2234	43.91	10.16	41.09	4.83	85.00	0.03	0.36
*C. dominula*	2165	45.13	9.52	40.60	4.76	85.73	0.05	0.33
*S. sinensis*	2168	43.82	10.75	40.68	4.75	84.50	0.04	0.39
*L. mitorrhaphes*	2157	42.42	9.92	42.88	4.78	85.30	−0.01	0.35
*E. dolicaon*	2146	43.20	10.02	41.71	5.08	84.91	0.02	0.33
*D. genutia*	2102	44.91	10.09	40.10	4.90	85.01	0.06	0.35
*B. mansfieldi*	2112	43.32	10.94	40.96	4.78	84.28	0.03	0.39
*C. benjaminii*	2132	43.76	9.66	41.70	4.88	85.46	0.02	0.33
*L. chandica*	2110	45.64	10.57	38.96	4.83	84.60	0.08	0.37
Control Region								
*C. andrei*	354	44.35	3.95	46.05	5.65	90.40	−0.02	−0.18
*C. trifenestrata*	327	43.73	3.36	46.18	6.42	89.91	−0.03	−0.31
*A. frithi*	333	42.64	3.90	46.55	6.91	89.19	−0.04	−0.28
*B. diniasalis*	336	42.86	1.49	51.79	3.87	94.65	−0.09	−0.44
*S. taiwanalis*	326	44.48	0.61	53.07	1.84	97.55	−0.09	−0.50
*O. semirubella*	310	43.87	0.65	50.97	4.52	94.84	−0.07	−0.75
*S. lubricipeda*	361	43.21	1.39	51.80	3.60	95.01	−0.09	−0.44
*N. fulgens*	363	44.90	1.10	50.69	3.31	95.59	−0.06	−0.50
*N. nigrisigna*	377	44.83	0.80	51.99	2.39	96.82	−0.07	−0.50
*G. argentata*	340	43.24	1.47	52.06	3.24	95.30	−0.09	−0.38
*C. dominula*	486	39.92	10.70	35.19	14.20	75.11	0.06	−0.14
*S. sinensis*	271	42.80	0.74	53.51	2.95	96.31	−0.11	−0.60
*L. mitorrhaphes*	369	46.07	1.63	49.05	3.25	95.12	−0.03	−0.33
*E. dolicaon*	509	43.22	3.54	45.19	4.32	88.41	−0.02	−0.10
*D. genutia*	440	44.55	1.14	51.14	3.18	95.69	−0.07	−0.47
*B. mansfieldi*	102	36.27	1.96	56.86	4.90	93.13	−0.22	−0.43
*C. benjaminii*	293	46.42	3.07	45.73	4.78	92.15	0.01	−0.22
*L. chandica*	413	44.07	2.91	48.91	4.12	92.98	−0.05	−0.17

**Table 5:** Mismatched base pairs identified in the predicted tRNA secondary structures of the *Cricula andrei* mitochondrial genome.

Trna	Amino Acid	Mismatch Type	Number of Mismatches
*trnM*	Met	G-U	2
*trnI*	Ile	G-U	2
*trnQ*	Gln	G-U	2
*trnW*	Trp	G-U	2
*trnC*	Cys	G-U	1
*trnY*	Tyr	G-U	2
*trnL2*	Leu	—	0
*trnK*	Lys	—	0
*trnD*	Asp	—	0
*trnG*	Gly	G-U	2
*trnA*	Ala	G-U	2
*trnR*	Arg	—	0
*trnN*	Asn	—	0
*trnS1*	Ser	G-U, U-U	2
*trnE*	Glu	—	0
*trnF*	Phe	G-U	2
*trnH*	His	G-U	2
*trnT*	Thr	—	0
*trnP*	Pro	G-U	2
*trnS2*	Ser	—	0
*trnL1*	Leu	G-U	2
*trnV*	Val	G-U	2

## Data Availability

The whole mitogenome of *Cricula andrei* is available in GenBank (Accession no. NC_084345), https://www.ncbi.nlm.nih.gov/search/all/?term=NC_084345 (accessed on 5 January 2024).
